# Literacy education for citizenship across the lifespan

**DOI:** 10.1007/s44020-022-00014-2

**Published:** 2022-06-06

**Authors:** Lesley Farrell, Tricia Eadie, Larissa McLean Davies, Carmel Sandiford

**Affiliations:** Melbourne Graduate School of Education, University of Melbourne, 100 Leicester St, Parkville, Victoria Australia

**Keywords:** Literacy, Citizenship, Technology, School English, Life course education

## Abstract

The task of English language and literacy education to define citizenship, and shape citizens, has rarely been more compelling or more challenging than it is today. Globally and nationally, our civic response to COVID-19 has catapulted us into a world where our rights as and responsibilities as citizens are being fundamentally re-negotiated at the same time as we come to rely on technologically mediated literate practice to connect a world that is more spatially and temporally separated than many of us have ever known it to be. We are challenged to remake our identities and commit to new kinds of personal and civic relationships—nationally and globally—as we try to navigate uncharted waters. Our focus here is on the role that literacy education plays in understanding and defining active citizenship in a turbulent context in which foundational literacy practices are transforming just as accepted understandings of active citizenship are under challenge. We direct our attention specifically to the distinctive role that literacy practice plays in the production of identities and relationships and consider new ways for literacy education to build active citizenship across the lifespan from early education through primary and secondary education through to workforce education.

## Introduction

Australia’s civic response to the COVID-19 virus has propelled us into a world that relies on technologically mediated literate practice to an extent that we could not have imagined prior to the pandemic. As good citizens, we worked from home when we could and socialised with our friends and family largely at a distance. The ligatures of citizenship became textual in character: video-conferencing peer-to-peer software platforms (like Zoom, FaceTime and Skype) became ubiquitous (the variability in Internet access becoming obvious) and the literate practices of toggling between the reading and writing needed to navigate the technologies and the face-to-face human interaction it enables became a ‘basic literacy’ with a speed we could not have predicted. In the space of 3 months, all of our most fundamental human activities—learning, working, caring and connecting—were, in large part and for most people, accomplished remotely and relied for their accomplishment on our capacity to exploit existing literacy practices, and to learn and generate new literacy practices, to connect us with people and technologies we needed to keep our lives in motion. Living with COVID-19 has taught us what these literacy practices make possible; it has produced new ways of being a student, teacher, worker, employer, parent, friend, carer etc., and created new relationships between all of these identities. It is unlikely that we will relinquish these nascent identities and relationships, even as we resume the old. Full citizenship, which encompasses all these identities and relationships, is being produced and expressed differently.

What are the challenges to citizenship in this brave new world? How can we fulfill our responsibilities to our fellow citizens and the next generation by providing education that supports active citizenship and prepares them to recognise and meet these challenges? These questions have profound implications for all sectors of education and for education across the lifespan. In this paper, we focus specifically on literacy, asking: What literacy practices and resources do we need to animate active citizenship in these new times? In addressing this question, we enter two distinct and connected arenas of febrile debate. Our two key terms, ‘literacy’ and ‘citizenship’, are hotly contested in both scholarly and contemporary political debate. Readers of *AJLL* will be familiar with contemporary debates around the term literacy so we will not rehearse them here: when we refer to literacy, we are referring to the practices of reading, viewing, writing, listening and speaking about text.

The term citizenship requires slightly more extended discussion. From a strictly legal perspective, the definition of citizenship is straightforward. The term refers to an individual’s membership of a category conferred by a nation state. Its use in scholarship and in public debate, however, is both more malleable and more contested (Young, 2004). Debates around definitions of the term citizenship centre partly on the emphasis placed on rights versus obligations, specifically on the tension between, and the relationships between, the individual, the State and the various communities to which the individual belongs or aspires to belong. For instance, when we use the term participation in relation to citizenship, what does participation mean? It can indicate a limited but fundamental ability to cast a vote, a passive ability to read and answer questions on a citizenship quiz, a technical ability to write a letter to the editor, the capacity of the individual citizen to conceive of themselves as someone who can initiate action in a political landscape, the obligation of a citizen to go to war in service of the collective, or the State. As social and political contexts are always changing, these relationships, and the identities with which they are associated, can never be static; they are always being renegotiated and redefined in the micro interactions of everyday life (Wan, 2011). In the field of education, options need to be open to recognise and embrace ‘cultural ways of knowing and diverse epistemologies’ that might otherwise be repressed (Tierney, 2018, p. 398). We do not have the space to pursue these debates in any detail here. For the purposes of our discussion, we adopt the settlement articulated in the Australian Curriculum—the capacity for individuals and communities to animate the ‘three components of citizenship – civil (rights and responsibilities), political (participation and representation) and social (social values, identity and community involvement)’ (ACARA, 2012). More than this, we understand active citizenship to be a practice; it is ‘what citizens do to improve the life experiences of themselves and others’ (Cranley et al., 2018, p. 2). Fundamental to our understanding of the practice of citizenship are the values of social justice, equity and human rights (Cranley et al., 2018). We know these terms take on new meanings in new times and as individuals and societies confront the enormous challenges that COVID-19 has brought into sharp relief, we believe it is crucial that literacy education actively works towards conceptualisations of literacy and citizenship that work for change across the lifespan. In articulating this goal, we acknowledge that literacy education has been, and still is, recruited to agendas that emphasise the obligation of the individual to the State, and, indeed, to diminish the capacity of the individual to assert any rights at all (Brandt, 2001; Crowther & Tett, 2011; Donald, 1983; Graff, 1979; Wan, 2011).

We are particularly concerned with the distinctive role of literate practice in ‘establishing, maintaining and repairing relationships. .. between people (often separated by geographical and temporal distance) and between people and technologies’ (Farrell et al., 2020) in all the domains of contemporary life. This is not a new concern for literacy studies. Literacy theory has always attended to the fundamental role of literate practice in shaping identity and relationships. Halliday’s interpersonal metafunction (1994/2004), for instance, offers a lens through which we can consider ways of enacting and evaluating roles and relationships through language across a repertoire of literate practices. Our particular focus here is in exploring the potential of literacy education across the lifespan in articulating and addressing the distinctive challenges to individual local, national and international citizenship in these turbulent times.

We begin by considering the earliest years of education paying particular attention to oracy, move to primary education and the role of the interpersonal, then secondary education and the role of the literary, and finally work-related education and the impact of the technologisation of work practice that powers the fourth industrial revolution. We conclude by considering the implications for public policy and specifically for initial teacher education.

## Language in early childhood as the first step towards citizenship

Children are born with an innate capacity to communicate, to develop a system of knowing and expressing themselves, which allows them to begin understanding the world around them. Language learning requires children to attend to, listen and imitate more proficient users. However, for most children, direct teaching of oral language is not required, as eminent linguist Noam Chomsky explains ‘nobody is taught language … in fact you can’t prevent the child from learning it’ (Chomsky, 1994). While children bring innate skills to learning, their language skills emerge through social interaction with caregivers in the context of the environment in which they live (Chapman, 2000). Increasingly, language skills are seen to be strongly affected by relatively small shifts in that environment (Asbury & Plomin, 2013). Conversational exchanges, the responsivity and reciprocity of caregivers, and social-emotional engagement are all essential ingredients in stimulating language learning environments. Studies of parent-child interaction reveal the importance of frequent, well-tuned, positive verbal interaction in supporting rapid language acquisition and children’s developmental pathways (Chapman, 2000).

Children develop an array of language skills each related to a different set of purposes (for example regulating behaviour, seeking attention and learning skills). In a few short years, children have large and varied vocabularies, can produce complex sentences, understand increasingly abstract concepts and generate stories. These various oral skills are differentially related to literacy (Snow, 1991). Importantly though, language establishes the foundations for later literacy, and as the two skills emerge, they become increasingly interrelated as the child ‘construes the code, and … construes the culture’ (Halliday, 1994, p. xxxi). Language is both the vehicle through which learning takes place, and the way in which children demonstrate their learning to others. In the preschool years, play is widely recognised as the leading context for the child’s acquisition of communication and collaboration skills. Play provides opportunities for children to express their personality, to be creative, curious and develop relationships. One of the most significant functions language and play serve in early childhood is to enable interpersonal relationships, that is the establishing, maintaining and repairing of relationships (Farrell et al., 2020).

Sociocultural theory (Vygotsky, 1978) asserts that children are motivated to communicate with others, making language fundamentally a social phenomenon. Children’s social and cognitive abilities mediate their path to competent language use. Halliday (1975) outlined more specifically the purposes or functions of language for young children. These functions help the child to satisfy physical, emotional and social needs. The functions of interest here include the *interactional*, where language is used to connect and form relationships with others and the *personal* where language is used to express feelings and build children’s identity and agency.

### A framework for language and literacy learning in early childhood

The role of early childhood education to support and scaffold language and literacy skills is articulated in the Early Years Learning Framework (EYLF) (DEEWR, 2009). The EYLF aims to provide a framework that supports early childhood educators to ‘extend and enrich children’s learning from birth to five years and through the transition to school’ (DEEWR, 2009). The EYLF describes young children’s learning and development outcomes in five domains: *children have a strong sense of identity; and a strong sense of wellbeing; children are connected with and contribute to their world; confident and involved learners; and effective communicators*.

With *effective communication* as one of its core learning and development outcomes, the EYLF recognises the importance of children’s communication and language abilities to provide the fundamental skills for their interactions with others, play-based experiences, and capacity to express their feelings and opinions. Equally, *children are connected with and contribute to their world* addresses children’s’ developing sense of citizenship. This is observable when children participate in decision-making and begin to cooperate and negotiate roles and responsibilities through play-based activities.

The EYLF articulates three important characteristics that emerge in children’s lives through their experiences with family, community and culture: *belonging, being and becoming.* Children’s interactions with others and participation in the world enable them to construct their sense of who they are and provide the first steps to citizenship (Harris & Manatakis, 2013). *Belonging* aligns closely with the ‘making relationships’ aspect of literate practice (Farrell et al., 2020). Children’s relationships and interactions with others are important to a strong sense of self and their connectedness to others, including family and educators. Effective communication skills mediate a child’s experience of belonging. *Being* encompasses children’s efforts to make meaning of the world in the here and now. Children’s experiences of reciprocal, responsive and sustained interactions build their sense of identity as a person who is valued, while also modelling and scaffolding their capacity to maintain and contribute to relationships (another of the literate practices). Finally, *Becoming* signals the substantial change that occurs as young children learn and develop, acknowledging that their understanding of the world, their capacity to negotiate or repair relationships, is an integral part of participating enthusiastically in society. Children’s use of language and their interactions with parents, peers, and educators provide them with a sense of who they are and that they are valued. When children’s ideas, questions and opinions are listened to, discussed and intrinsically respected, confidence and participation flourish.

In addition, the United Nations Convention on the Rights of the Child (UNCRC) (United Nations [UN] 1990, Article 13.1) states that all children have the right to freedom of expression, and to seek, receive and impart information. This view of the child as capable and having agency is aligned with contemporary thinking of childhood, here children are not only viewed as capable of expressing their views but have the right to do so, including the right to participate in the cultural and civic activities of their communities.

The capacity of children to make choices, to voice opinions and to be aware of the needs of others is part of their developing sense of identity. When children persist (often in the face of challenges), to negotiate and share, and recognise the achievements of themselves and others, they are developing the skills of active citizenship and participation in community. Children are learning about personal beliefs that build and shape their identity. Throughout, language and sustained interactions mediate this learning. Positive experiences and attitudes are critical to building competent communicators and citizens for now and the future, ensuring children’s strong sense of identity, wellbeing and motivation for life-long learning. The foundations of which are built through children’s earliest communication skills and language use in the first few years of life.

## Writing into citizenship in the primary years

From the initial development of a protolanguage through to learning the mother tongue, meaning ‘consists in simultaneously construing experience and enacting interpersonal relationships’ (Halliday, 1993, p. 101). As children transition from the early years of learning, where belonging, being and becoming frame their learning experiences, to later experiences in the primary school where notions of themselves as social beings and agents become more distinct, to secondary school where the study of literature might shape notions of empathy essential to establish, maintain and repair relationships, layering an interpersonal lens on texts and meaning is essential. Halliday’s theory of metafunctions (Halliday, 1994/2004) provides a principled means to understand how language performs three functions simultaneously: ideational (what the text is about and logical connections within), interpersonal (how interactions and relationships are created through texts) and textual (how texts are shaped and organised). While the three occur simultaneously, a focus on the interpersonal is most relevant as we examine ways learners can be supported to enact their responsibilities as citizens under ever-changing conditions.

A significant challenge in the primary years is the move from spoken to written language where students need to express their ideas and thoughts in more written-like language (Christie, 2005). In the context of school written language, children are quite often judged on this capacity, both formally and informally, and assessed on their literateness as distinct from their ‘languaging’. While this consideration is not new, ways in which language enables the individual to communicate in meaningful ways continue to be an important concern from an academic and interpersonal perspective; it impacts on how students manage the work of schooling while at the same time establishes their place as contributing citizens. How these academic and personal worlds are revealed through language choices and literate practices requires understanding not only of the different modes of communication, but also the systems which enable the meanings.

Building a repertoire of literate practices across the years of schooling should not be confined to a prescribed set of practices required for school learning; rather, understanding interpersonal systems of language choices and how they work to establish relationships and enable the expression of thoughts and opinions opens up the repertoire of possibilities (Martin & White, 2005). With a focus on ‘establishing, maintaining and repairing relationships’, an interpersonal lens on meaning offers interactive semiotic resources. One such system of choices is the Mood System where patterns of interaction through declarative, interrogative or imperative statements are presented. Another system, Modality, offers choices that span ‘degrees of obligation and probability in the territory between yes and no’ (Macken-Horarik et al., 2018, 143) and is realized, through modal verbs, adverbs, adjectives and nouns. Within the larger system of Appraisal (Martin & White, 2005), Attitude is principally concerned with the expression of feelings (Affect), judgements of human behaviour (Judgement) and the evaluation of the quality of things (Appreciation), all of which might be amplified or softened through Graduation. While these interpersonal resources, and others, are used across of range of texts for different purposes, here we emphasise their application to acts of persuasion where, in the development of argument, ‘resources from these sub-systems of Appraisal can be seen as roughly relating to appeals to Pathos (Affect), Logos (Appreciation) and Ethos (Judgement)’ (Macken-Horarik et al., 2018, p. 191). Control over these rhetorical ‘particulars’ for learners is as important in civic life in the twenty-first century as in ancient Greek times to support educational aspirations and fulfillment of well-informed citizenry (Macken-Horarik et al., 2018).

From a very early age, children are exposed to and engage in a myriad of persuasive forms as they demand and argue about their preferences of immediate needs, wants and expectations, often quite bluntly. Through the primary school years, students need to be apprenticed to developing ways to vary the force of their position, adopting a more measured, compassionate and nuanced stance which encompasses cultural, social and linguistic diversity and values. In doing so, learners can be supported to move from what might be ‘boring’ arguments reliant on top-level structure to more engaging persuasive texts (Love et al., 2014, p. 49). Across the family of persuasive texts (e.g. expositions, discussions, advertisements), patterns of interactions require a familiarity with the way suggestions, requests and demands can be made, and when one choice is more appropriate than another in seeking the desired outcome. For example, the inclusion of rhetorical questions at strategic points in an argument can be used to engage the reader, or to expand or contract argumentative positions (Derewianka, 2011, p. 114). In contrast, blunt imperative statements can work to alienate an audience. Similarly, judicious choice of modality is very much dependent on an understanding of personal interactions through a sophisticated use of ‘hedging (e.g., *may*, *partially*, *virtually*, *perhaps*) to show humility and politeness in arguments, and to lower feelings of opposition among readers or addressees’, a skill often overlooked or underdeveloped (Mills & Dooley, 2014, p. 33). Hence, whether to include high, medium or low levels of modality, and at which points in the text, involves selection of resources which open up or close down the extent to which possibilities might be considered.

Within Appraisal, Attitude expands the repertoire for enabling the expression of emotions or feelings about an issue, casting judgements about human behaviour through critique or praise, and voicing the qualities of things, all of which might be softened or amplified through Graduation. In her address to the United Nations, Greta Thunberg (2019) makes use of overt and highly amplified attitudinal choices, one reason her arguments appear at the same time both compelling to her peers and alienating to the adults at which her rhetoric is directed. In one sentence, ‘We are in the beginning of a **mass extinction**, and **all** you can talk about is **money** and **fairy tales of eternal economic growth**’, Greta clearly asserts her quite negative judgment of those not heeding warnings of climate change impact through explicitly inscribed attitude, leaving little room for a differing point of view. What she does here, quite strategically, is establish two kinds of relationships: one of equality, mobilising her right and that of her peers, to take a stance and be heard on a global issue; and the other, confronting older citizens she deems responsible.

There is no question about the ‘centrality of argumentation to learning and civic life’ (Chen et al., 2021, p. 46). Establishing an understanding of the learner’s world and the capacity to engage critically with audiences to express their points of view is essential for the learner in becoming an active and responsive citizen. Across the life span, attention to interpersonal linguistic resources becomes increasingly important as the nature of literacy changes and as interacting with others shifts ‘from the unmediated expression of personal feelings and emotion through to more judicious evaluation of behavior and phenomena based on institutionalized norms’ (Christie & Derewianka, 2008, p. 15). Explicit attention to these interpersonal systems of choice and how they might be co-patterned across a text empowers students’ potential actions as informed and social citizens—taking part in debates, taking a stance, challenging ideas while at the same time building relationships, establishing a sense of belonging and contribution, persuading across differences, maintaining equality, showing optimism in troubled times, skills central to becoming and being an active citizen.

## Understanding citizenship through literary education in the secondary years

Literacy education in the Early and Primary years of education becomes, in the secondary years, both located in Subject English and also embedded in the disciplines. Literacy disciplinary knowledge developed, for example through science, history and art undoubtedly contributes to the development of students’ understanding of citizenship, and the choices and life they will lead outside the secondary context, in the world of work and in any tertiary study they may undertake beyond the secondary school years. Indeed, Civics and Citizenship is a core subject as part of the Humanities suite of subjects in both National and State curricula (ACARA, 2012; VCAA, n.d.; NSW DET, n.d.). Yet, it is arguably subject English, the single curriculum area mandated across the stages of schooling, that is allocated (along with mathematics) the greatest amount of time in the curriculum^1^ and mandated in most States in the post-compulsory years, where general literacy and citizenship is understood to reside. This is reflected in the organisation of the curriculum itself: for example the *Australian Curriculum* identifies literacy as a cross-curriculum priority (ACARA, 2012), and more recently has provided literacy progressions for all discipline areas but does not locate literacy centrally in the disciplinary curriculum content. English in the *Australian Curriculum*, however, has a key strand called ‘literacy’. This distinction is amplified in the authors’ home State of Victoria, where literacy is understood to be the responsibility of all secondary teachers but is both located and assessed in English (VCAA, n.d.).

Along with general literacy responsibilities, secondary English has the related responsibility for preparing future citizens for productive lives. Writing from the British context, Bela and Hopkins note that the history of Subject English (SE) is inextricably bound, as far as governments are concerned, with citizenship education:

‘… more than simply resembling citizen-ship education, SE [school English] emerges in the first instance as a form of highly normativising civic education. Indeed, English education generally has at its ‘core’, so to speak, a concern with the moulding of ‘good’ (that is, desirable) citizens, particularly in the face of an expanding electorate.’ (Belas & Hopkins, 2019, p. 321).

While language, literature and literacy all form core components of subject English in Australia (ACARA, 2012), it is historically and primarily literature, in our country, and in other western nations, that is charged with the work of developing a future citizenry (Hunter, 1988; Patterson, 2008). The history of English education in British and colonised contexts shows consistent arguments for the capacities of literature to enable students to access experiences beyond their own, become ‘spectators’ (McLean Davies et al., 2022; Squire, 1968) of other lives and ways of being, and through this develop empathy (Mangen et al., 2018), expand their own narratives and reconsider social possibilities for the future (Dale, 2012). Like previous scholars, in recent times, researchers concerned with developing a global, empathetic citizenry have turned to the possibilities of diverse literary works and the teaching of literature in schools (Holmes, 2019) and universities (Saleem & Ilyas, 2019).

Perhaps because of its apparent transformative potential, the teaching of literature itself remains both a prime motivator for individuals to become English teachers (Goodwyn, 2012; Manuel & Hughes, 2006), and much contested aspect of secondary English, as ongoing debates about text selection attest (Hastie & Sharplin, 2012; McLean Davies, 2012; Yates et al., 2019). In a subject which both powerfully contributes to the formation of future citizens, but where content (in the form of literary works) is not fixed, professional and public debates about text selection in school English can be understood, at least in part, to reflect concern with the dispositions, alliances and concerns that students, as future citizens, will develop through the study of literature.

The use of literature to support the development of a homogeneous, law-abiding, moral and aspirational citizenry was famously championed by Matthew Arnold, who was an Inspector of schools, academic and poet in England in the nineteenth century. This position has routinely been attacked by Marxist critics such as Eagleton (2008), and personal growth advocates such as Dixon (1975). Although motivated by different theoretical and pedagogical positions, both take issue with the cultural heritage model of English that supports the development of a particularly compliant middle-class population. Recent scholarly criticism of policy directions in England regarding the teaching of literature in school English has argued that the return to and emphasis of canonical literature in the school English curriculum has impoverished the ethical and transformative potential of this school subject in contemporary times (Belas & Hopkins, 2019).

As a result of contemporary concerns about the deployment of English and literature to animate conservative and compliant citizens, English teachers have not necessarily privileged citizenship discourses, preferring to emphasise individual and personal growth models of working with texts (Frawley, 2016; Yandell, 2016), or critical literacy approaches, which, while interrogating the way a text is shaping society, have not always extended to action regarding how injustice might be addressed (Janks, 2009). Although citizen formation has not always been dominant in the discourses on the English classroom, however, studies in the Australian context have shown that literature has reinforced, both explicitly and implicitly, through a preference for canonical British and North American texts colonial and masculine paradigms of nationhood (McLean Davies & Buzacott, 2018; McLean Davies & Martin, 2017). In this context, the mandating of Australian literature, by the Australian Curriculum and Reporting Authority since 2008, can be seen as a tangible effort to address this issue and open up dialogue about a more diverse understanding about Australian citizenry and society.

It seems clear that, in the context of global pandemic, social and environmental crises, it is time to rethink the ways in which secondary English contributes to citizen formation, and the ways we understand, speak, read and write about ourselves in the world. Literature, and the telling and receiving of stories, plays a key role, here, in the way we enact the present, and imagine the future (Phillips et al., 2022). The Australian Black Lives Matter protests, which focused on unexamined and unacceptable Indigenous deaths in custody, brought renewed attention to the urgent need for rethinking citizenship discourses: what it is to be an Australian citizen, and what constitutes national identities. It became clear to many, perhaps for the first time, that Indigenous stories need to be prioritised in a country that continues to be shaped and governed by enduring, colonial conceptions of nation. Various media drew these personal stories to public attention and members of the public purchased Aboriginal texts of both fiction and nonfiction, resulting in publishers and bookstores being unable to meet the sudden and increased demand (Harris, 2020; Kembrey, 2020; McLean Davies et al., 2021a).

Rather than reinforcing colonial notions of citizenship through the dominant study of canonical literature, we have an opportunity, as we embark on the next decades of the twenty-first century, to rethink the work of literature in subject English, beyond individual engagement and personal response and to enact a ‘literary literacy’ (Green, 2014). This, of course, means more than listing books considered to be ‘representing’ diverse perspectives for study. Research has shown that listing texts will not necessarily change local text selections practices or institutional reading priorities which depend on generation of intertextual networks which often privilege canonical texts (Bliss & Bacalja, 2020; McLean Davies et al., 2021a, b). Rather, we need ongoing commitments, first, to acknowlede the key role and potential of literature in the formation of twenty-first-century citizens, and second, to ensure all students have the opportunity to expand their experiences and understandings of one another through text so that they can meet the demands of contemporary citizenship and improve life justly, for themselves and others.

## The literate practices of citizenship at work across the lifespan

Literate practice and work practice are inextricably linked to public perceptions of productive working identities. The claim that the literacy skills of the contemporary workforce are not keeping up with the literacy demands of the contemporary workplace is a familiar refrain in public debate and has been for decades (see for instance AiG, 2018; Productivity Commission, 2018; Shomos & Forbes, 2014). While the relationship between literate practice, work practice and the literate practice of productive citizenship is often asserted, it is, however, more complex and more problematic than it might seem to be from public rhetoric. The association between work and literacy is not new; even the Romans needed *some* workers (who could read and write in order to keep abreast of the economic activities of the Roman Empire, but they were most often slaves). The broader requirement for *more* workers to be literate emerged around the time of the first Industrial Revolution, around about the time that literacy was becoming associated with political participation.

During the First Industrial Revolution, however, it became clear that steam-powered automation relied on precisely followed written instructions—and thus it relied on a workforce that could read and, more rarely, write. Governing classes struggled with the conundrum that a literate workforce could power both Industrial Revolutions (an advantage from their point of view) and Political Revolutions (dangerous from their point of view) (Donald, 1983; Farrell et al., 2020). A literate workforce could read and comply with written instructions, but it could also (both overtly and covertly) communicate revolutionary ideas, provoke revolutions and strategise violent uprisings to assert claims to mass citizenship as the French and American Revolutions were demonstrating. From the perspective of the governing classes, the challenge for mass education was to develop a workforce that was simultaneously literate and compliant. In order to achieve that aim, early formal education concentrated on reading and ignored writing. However, it proved to be very difficult to control literate practice once reading and writing had gained a foothold, and literacy became a feature of both work and citizenship.

We are now at the dawn of the Fourth Industrial Revolution, and this revolution, too, is making new demands on literacy and presenting new challenges to citizenship. The Fourth Industrial Revolution is marked by emerging technology breakthroughs in a number of fields, including robotics, artificial intelligence, nanotechnology, quantum computing, biotechnology, the internet of things, 3D printing and autonomous vehicles. Essentially, the fourth industrial revolution is about the convergence of these new technologies—technologies which can communicate with each other and learn from each other. This is changing what work is available, how that work is organised and how the work is accomplished. It is now the case that anything, anything at all, that can be described by a set of steps or actions—anything routine—can be automated. This goes beyond manufacturing, where it is well advanced, to include major tasks associated with professions previously considered to be impervious to automation, like accounting, law and scientific research; high status occupations which include highly specialised, and highly rule-governed practices. This is not to say that these work tasks *will* be automated; political, social and economic factors contribute to specific decisions to automate specific tasks. It is to say they can be, and probably will be, when politically, economically and socially hospitable conditions arise.

Another outcome of this technological revolution is that work practice is no longer linked to place in the ways that it once was. The COVID-19 pandemic, with its urgent injunctions to work at home, has spectacularly accelerated our move from work*places* to work*space*s—dynamic, fluid, often transient, working units defined and bounded by regular, routine information and communications technology routes (Farrell, 2001, 2006).

These moves, away from routine work and towards remote work, have changed the kinds of literate practice that contemporary work requires. The term Literacy 4.0 is used as a shorthand to capture the sense in which work practice has become literate practice:

A distinguishing feature of Literacy 4.0 compared with previous iterations of workplace literacy is, therefore, the increased focus on the establishment, maintenance and repair of working relationships between people, technologies, organizations and the elements of globally distributed value chains. The increased focus on the literate practices associated with making and maintaining connections arises directly from the defining feature of the Fourth Industrial Revolution: technological convergence (Farrell et al., 2020).

Like other work practices, the literate practices of work are moving away from the routine (machines can now manage most routine forms of literate practice) towards distinctively human, technologically enabled, customised forms of literate engagement to achieve problem solving, innovation and the establishment, maintenance and repair of all the relationships that make up contemporary workspaces. These kinds of literate practices require a distinctive approach to literacy education across the lifespan. As far as work-readiness is concerned, it is no longer plausible to confine literacy education to early childhood education and schooling. As work practice and work organisation changes in response digitisation, we will all need professional education across the lifespan that supports us in learning new ways of reading, writing, listening and speaking about the multimodal texts that constitute our working lives.

These developments in the digitisation of work, and especially changes to the ways that work is organised, make new demands on the literate practices of active citizenship. The trend towards casual work arrangements, often although by no means always managed through general companies like Airtasker or more specialised companies like Script using peer to peer software, has eroded working conditions like sick leave and mandated occupational health and safety requirements, leaving workers—from the least skilled to the most skilled—to fend for themselves (Corbel et al., 2021). To protect themselves in these circumstances, individual workers need to learn to read contracts and other documents, and to write them, in ways that protect them from exploitation and harm. The implications of the digitisation of work are not confined to the individual worker. As a society, we are currently experiencing trends towards insecure and underemployment, especially in relation to young people, and increasingly precarious work arrangements for everyone. The economic consequences of COVID-19 are only exacerbating trends that have been evident for decades. There is less secure work available, the knowledge and skills people need to do the work are less predictable and the lifespan is no longer marked by the professional and social milestones that we have taken for granted for decades (Chesters et al., 2019). These challenges must be dealt with collectively, with a new model of active citizenship animated by sophisticated literate practices which can attend to the establishing, maintenance and repair of all kinds of relationships—local, national and global.

## Conclusion

At its core, the best literacy education (including the development of oracy) foregrounds the interpersonal—the establishment, maintenance and repair of relationships—across all the domains of human experience, whether it be to express ideas and opinions, tell and then write our own stories, and through the literary, inhabit the lives and stories of others.

Since its inception, literacy education has been shaped by productive tensions: the tension between the development of a skilled (and compliant) workforce on the one hand and the development of a robust citizenry on the other; and the tension between the inculcation of respect for established knowledge and traditional relationships on the one hand and the capacity to challenge established knowledge and forge new relationships between people and between people and institutions on the other. In that sense, the challenges we face are no different from the challenges faced by those who have gone before us. We are, however, confronted with circumstances that are distinctively daunting and urgent. The convergence of global climate crises, global health crises and the profound social, political and economic upheavals generated by technological transformations of work challenge the relationships between people, technologies and institutions that form the foundations of our communities and our institutions. Literacy practices are the ligatures that establish, maintain and repair these relationships, the relationships on which active citizenship, positive social, political and economic transformation depends.

What can we, as educators, teacher educators and public policy makers, do to make and sustain an environment in which the literacy practices which generate, support and protect these ligatures are strengthened? First, we can break down the traditionally siloed education sectors to promote literacy education across the lifespan. Effective literacy education for citizenship starts at birth and does not end with formal education but extends through every stage of our adult lives. Second, we can take what we have learned about literate practice from past research and theorising on literacy and test it against the contemporary and emerging lived experience of people unlike ourselves. Specifically, we need to commit to engaging with new, literacy-saturated workspaces and other newly emerging literacy-saturated social, political and economic institutions and learning about how they work and what they mean for the lives of our students. Finally, we can attend to the stories we tell and the stories we ask our students to write, recognising that these are the resources that will help our students imagine new worlds, new ways of being and new ways to relate with each other.

## Footnotes

1 The Humanities and Sciences are also allocated substantial amounts of time; however, both of these discipline areas include multiple subjects (VCAA, n.d.).
